# Recognition of Depression and Anxiety among Elderly Colorectal Cancer Patients

**DOI:** 10.1155/2010/693961

**Published:** 2010-03-23

**Authors:** Amy Y. Zhang, Gregory S. Cooper

**Affiliations:** ^1^Frances Payne Bolton School of Nursing, Case Western Reserve University, 10900 Euclid Avenue, Cleveland, OH 44106-4904, USA; ^2^Division of Gastroenterology, School of Medicine, Case Western Reserve University, 10900 Euclid Avenue, Cleveland, OH 44106-4904, USA

## Abstract

This study investigated the ICD-9 diagnostic rates of depressive and anxiety disorders, including major depression, neurotic depression, adjustment disorder with depressed mood, depressive disorder NOS (not elsewhere classified), and anxiety states, among elderly Medicare beneficiaries (age ≥ 65) who received a colorectal cancer diagnosis between 1998 and 2002 in U.S. The Seer-Medicare data, representing 14–25% of the U.S. population, was used to examine ICD-9 diagnostic rates of depressive and anxiety disorders among 56,182 colorectal cancer outpatients and 265,382 noncancer outpatients, respectively. The findings show that the ICD-9 diagnoses ranged from 1.5% to 1.8% for depressive disorders and 0.8% to 1.2% for anxiety states in the colorectal cancer outpatients, and from 2% to 2.5% for depressive disorders and 1.1% to 1.5% for anxiety states in the noncancer outpatients over five years. More than 70% of colorectal cancer outpatients with a depressive diagnosis were diagnosed for depressive disorder NOS. The findings suggest that the difficulty in recognizing depressive symptoms in colorectal cancer patients may contribute in part to the low ICD-9 diagnostic rates of depressive disorders. They call for research attention to the investigation of depressive symptoms for improving the recognition and treatment in this patient population.

## 1. Introduction

The prevalence of major depression is estimated to be 1–4% among the elderly but as many as 16% of the elderly experience significant depression symptoms in the U.S. [1]. The Epidemiologic Catchment Area (ECA) study reported 1.4% lifetime prevalence and 0.9% 12-month prevalence of major depression in community residents aged 65 and older [2]. Depression in the elderly is concomitant with comorbidity. Cancer is one of the common diseases in the aged population. The prevalence of depression in cancer patients ranges from 1.5% to 58%. It is commonly held that 20% to 25% of cancer patients manifest symptoms of a major depressive episode at some point in time [3]. 

Colorectal cancer is the third most common type of cancer in the United States, projected to affect 146,970 men and women in 2009. Given the steady decline in mortality rates of colorectal cancer, 58% patients would live more than 10 years after initial diagnosis [4]. Studies that used the Center for Epidemiologic Studies Depression Scale (CES-D) as a screening tool have reported that 18–24% of newly diagnosed colorectal cancer patients likely have clinical depression with a CES-D score ≥16 [5]. For those who survived colorectal cancer for more than five years, 14% were found to be depressed [6]. Not surprisingly, African-American colorectal cancer patients were found to be more depressed than white patients on the CES-D [5]. Female colorectal cancer patients were found to be more depressed on the CES-D than male colorectal cancer patients [5]. There is also evidence that younger and newly diagnosed patients experience greater distress than older or previously diagnosed patients for colorectal cancer [7]. 

Despite evidence of prevalent depression and anxiety among colorectal cancer patients from survey studies, information about the clinical identification of depressive and anxiety disorders among colorectal cancer patients is scarce. This information is critically important because it provides baseline data on how depression and anxiety are recognized and treated in the current medical system with regard to a cancer diagnosis. It is especially important for the elderly population in that 64.5% of new colorectal cancer diagnosis occurs among persons aged 65 years and older [8]. The striking health disparities also speak for the need of a careful examination of the diagnosis of depression and anxiety in African American patients. This information will help us better to understand how to detect and treat depression and anxiety, which is critical to the quality of life of colorectal cancer patients. 

Therefore, this study examined depressive and anxiety diagnoses among elderly Medicare beneficiaries with colorectal cancer (age ≥ 65). It aimed to address three questions: (1) what are the diagnostic rates of depressive and anxiety disorders in elderly colorectal cancer patients in the Medicare system? (2) Are there differences in the diagnostic rates between noncancer outpatients and the newly diagnosed or the previously diagnosed elderly colorectal cancer patients? (3) Are there differences in the diagnostic rates across age, gender, and ethnic groups? 

## 2. Methods

The study used the linked Surveillance Epidemiology and End-Results (SEER)-Medicare data [9]. The SEER program is the largest cancer surveillance system in the U.S., sponsored by the National Cancer Institute. It collected information on newly diagnosed cancer cases that occurred in persons residing in nine states, which represented 14% of the US population in 1998 and 1999, and 25% between 2000 and 2002. The SEER data provided information on the time and type of cancer diagnosis for this study. The linked Medicare data contains medical claims for persons aged 65 or older and was used to provide psychiatric diagnosis for colorectal cancer patients identified from the SEER. Patient eligibility included a new diagnosis of colon and rectum cancer, age of 65 and older at the time of this diagnosis, and 12-month full enrollment in Part B Plan during a study year. Patients who had partial participation in Part B Plan were not included because they might have received a psychiatric diagnosis elsewhere, thus confounding the study outcome. Medicare participants who were enrolled only in Part A Plan (inpatient service) were excluded due to a lack of information about their medical care in the outpatient setting. Further, the claims for HMO enrollees in the Medicare system were not included in SEER-Medicare data and therefore HMO enrollees were excluded from the study. The exclusion criteria reduced the study sample size by approximately 43%. In addition, the SEER program collects information on a random sample of 5% of persons without a cancer diagnosis residing in the survey area. The same eligibility criteria, except the cancer diagnosis, were used to retain patients without cancer. This noncancer subsample was used as a comparison group. 

The study examined depression and anxiety diagnoses in persons that were diagnosed with colorectal cancer between 1998 and 2002. The SEER data contained a total of 56,182 newly diagnosed patients over the five study years, including 7,308 people in 1998, 7,046 in 1999, 13,811 in 2000, 13,819 in 2001, and 14,198 in 2002. The noncancer subsample totaled 265,382 people, with 142,696 to 174,372 enrolled in the Medicare system each study year.

 In the SEER data, a colorectal cancer diagnosis may indicate an initial occurrence of cancer or second primary cancer, which may be associated with different levels of psychological distress [10] and possibly different diagnostic rates of depressive or anxiety disorders. Thus, we identified all the patients who received a colorectal cancer diagnosis during a given study year between 1998 and 2002. The patients who received this colorectal cancer diagnosis as a primary diagnosis (the first lifetime cancer diagnosis) in a given study year were identified as newly diagnosed. The patients who received this colorectal cancer diagnosis as a secondary cancer diagnosis in a given study year or a diagnosis of any cancers prior to the given study year were identified as previously diagnosed. In the study, the full sample of colorectal cancer cases, the newly diagnosed cases and the previously diagnosed cases were obtained annually from the SEER data and examined separately for rates of psychiatric diagnoses. 

The diagnosis of depressive and anxiety disorders was obtained from the Medicare data. The Medicare data used the International Classification of Diseases (ICD-9) codes for diagnoses of mental disorders. The ICD-9 has a total of four diagnostic categories of depressive disorders: major depression (codes 296.2, 296.3), depressive disorder NOS (not elsewhere classified) (code 311), neurotic depression (code 300.4), and adjustment disorder with depressed mood (code 3090). All of the four diagnoses and the diagnosis of anxiety states (code 300.0) were examined in this study. Each Medicare claim has a maximum of 10 diagnostic fields. All the 10 diagnostic fields were counted and a diagnosis was assigned when an individual was diagnosed at least once for a disorder during a given year. Demographic information on age, gender, and race that was reported along with depression and anxiety diagnoses in the Medicare data was used for analysis. 

The data analysis was conducted using the Statistical Analysis Software (SAS 9.1). When calculating the rates of psychiatric diagnoses, the total number of colorectal cancer patients in the full sample, the newly diagnosed sample, and the previously diagnosed sample were used as the denominator separately, whereas the number of colorectal cancer patients who had a psychiatric disorder in a sample was taken as the numerator. The rates were calculated by dividing the number of colorectal cancer patients having the same psychiatric disorder by the total number of colorectal cancer patients in a sample. The rates were computed for each sample annually. The same approach was applied to compute the diagnostic rates for the noncancer subsample, wherein the number of noncancer persons with a psychiatric diagnosis was divided by the total number of noncancer persons in a given study year. 

Fisher's Exact test was performed to compare the newly diagnosed and the previously diagnosed patients with the noncancer controls, respectively, in the diagnostic rate. Further, we compared the diagnostic rates of depressive and anxiety disorders by age, gender, and ethnicity for the cancer sample to examine associations between a psychiatric diagnosis and demographic factors. Age was grouped into three categories: young-old (age of 65–74); mid-old (age of 75–84); and old-old (≥age of 85). Mid-old and old-old groups were compared separately with the young-old group that might be vulnerable to depression. Race was grouped as whites, african Americans, and “other” (Asians, Hispanics, etc.). The white group was compared with the African American group and the “other” group. The Fisher's Exact Test was performed for pairwise group comparisons on the diagnostic rates using the full sample of cancer patients.

## 3. Results

Demographic information of 56,182 colorectal patients diagnosed between 1998 and 2002 is presented in Table 1. Overall, these patients included slightly more women (53.4%) than men. Forty three percent of them were between the ages of 65 and 74 (young-old), 41% were 75 to 84 years old (mid-old), and 16.3% were 85 years or older (old-old). The majority (84.7%) was white and 7.7% were African American. The patients primarily had early stage cancer (I–III) with 9.8% having distant metastases (stage IV). The noncancer sample of 265,382 Medicare enrollees, on the other hand, had more women (63%) than men (37%) and a similar distribution in age and race. 42.5% Medicare enrollees were between the ages of 65 and 74, 39.6% were 75 to 84 years old, and 17.9% were 85 years or older. The majority (82.8%) was white and 7.4% were African Americans.

### 3.1. Diagnostic Rates of Depressive and Anxiety Disorders

Table 2 presents the diagnostic rates of depressive and anxiety disorders for three samples: the full sample of cancer patients, the newly diagnosed sample, and the previously diagnosed sample. For the full sample, the diagnostic rates of depressive disorders varied from 1.5% to 1.8% and the diagnostic rates of anxiety states varied from 0.8% to 1.2% between 1998 and 2002. This indicates that one or two out of 100 colorectal cancer patients were diagnosed for depressive disorders and anxiety states in any given study year. Further, during the same period, the diagnostic rates of depressive disorders were 1.4% to 1.9% for patients without a prior cancer diagnosis and 1.4% to 2.5% for patients with a prior cancer diagnosis. The diagnostic rates of anxiety states were 0.9% to 1.3% for patients without a prior cancer diagnosis and 0.6% to 1.2% for patients with a prior cancer diagnosis between 1998 and 2002. Despite an apparently higher trend of diagnostic rates in patients with a prior cancer diagnosis, we did not observe significant differences between the newly diagnosed and the previously diagnosed cancer patients in the rate of depressive and anxiety disorders using the Fisher's Exact test. 

The breakdown of the diagnosis of depressive disorders by category shows that the diagnostic rate was highest for depressive disorder NOS, varying from 1.1% to 1.4% over time, and lowest for adjustment disorder with depressed mood, varying from 0.04% to 0.1% over time. Diagnostic rates for major depression (0.2% to 0.4%) and neurotic depression (about 0.1%) were intermediate across time. The proportion of various depressive diagnoses ranged from 71% to 82% for depressive disorder NOS, 13% to 26% for major depression, 7% to 10% for neurotic depression, and 2.7% to 5.5% for adjustment disorder with depressed mood over time.

### 3.2. Comparison of Diagnostic Rates with the Noncancer Sample

**Figure 2:**
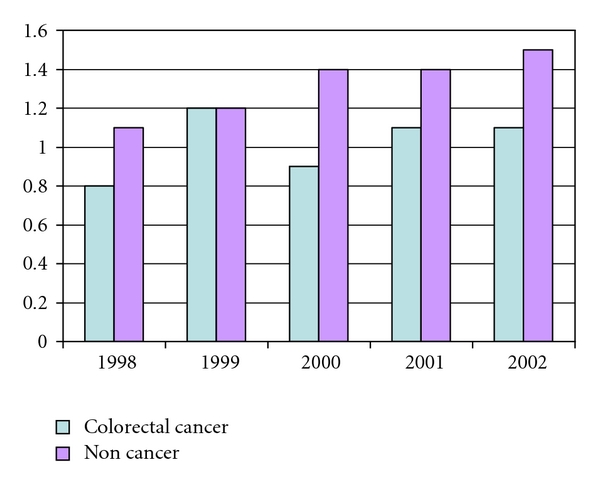
Comparison between a Noncancer Sample and a Colorectal Cancer Sample: Anxiety State.

The diagnostic rates appeared to be higher in the noncancer sample than in the colorectal cancer sample as shown in Figure 2. They were 2%, 2.1%, 2.3%, 2.3%, and 2.5% for depressive disorders, 1.1%, 1.2%, 1.4%, 1.4%, and 1.5% for anxiety states from 1998 to 2002. The diagnostic rate was highest for depressive disorder NOS (1.4% to 2%) and lowest for adjustment disorder with depressed mood (0.02% to 0.1%), 0.4% for major depression and 0.2% for neurotic depression over time. The proportion of depressive diagnoses was similar to the cancer sample, ranging from 71% to 79% for depressive disorder NOS, 16% to 23% for major depression, 8.9% to 10.8% for neurotic depression, and 0.9% to 4.1% for adjustment disorder with depressed mood over time. 

The newly diagnosed sample had a significantly lower rate of depressive disorders in four of five years (1998, 2000–2002) and a significantly lower rate of anxiety disorders in three of five years (2000–2002) than the noncancer sample. We did not detect a significant difference between the previously diagnosed sample and the noncancer sample in the rates of depressive and anxiety disorders over time.

### 3.3. Comparison of Diagnostic Rates by Demographic Factors

Table 3 presents diagnostic rates by age, gender, and ethnicity for the full sample. The higher rates of depressive disorders were observed in older, female, and white patients. As Table 3 indicates, the diagnostic rate of depressive disorders was significantly higher for patients aged 85 or older than for younger patients (age of 65–74) in four of five years (*P* ≤ .05), except 2002. Significantly more female patients were diagnosed with depressive disorders (*P* ≤ .001) or anxiety states (*P* ≤ .01) than male patients in all years. White patients were diagnosed for depressive disorders at a significantly higher rate than African American patients in years of 1998 and 2001 (*P* ≤ .05) or patients with other ethnic status in 1999 (*P* ≤ .001). 

## 4. Discussion

An important finding of this study is that the ICD-9 diagnostic rates of depressive and anxiety disorders were low for this large cohort of colorectal cancer patients. The diagnostic rate varied from 1.5% to 1.8% for all depressive disorders combined and 0.8% to 1.2% for anxiety states across the study years. The diagnostic rate of major depression alone (0.2–0.4% for the cancer sample and 0.4–0.45% for the noncancer sample) is also lower than the 12-month prevalence of 0.9% in the ECA study that used DSM-III diagnostic criteria. The time trend of the diagnostic rates was relatively stable, demonstrating the reliability of these findings. When we included HMO enrollees and partial Part B enrollees in the analysis, the diagnostic rates in cancer patients were even lower, suggesting that the study findings were not influenced by the exclusion criteria. 

Few studies have examined the rates of ICD-9 psychiatric diagnoses in the Medicare population. A study of Medicare beneficiaries in Tennessee [11] reported that the ICD-9 diagnostic rate of mood disorders was 2.9% for women and 1.0% for men between 1991 and 1993, which approximates the findings of the present study. This suggests that the rate of ICD-9 psychiatric diagnosis is generally low because its diagnostic criteria are different from DSM-III and more stringent than the survey scales. The factors pertaining to how the ICD-9 diagnoses are coded and reported may also contribute to the low rate of ICD-9 diagnoses [12]. There is also a possibility that some colorectal cancer patients were treated with psychiatric medications without a formal diagnosis. However, the Seer-Medicare data do not contain information on physician prescription, precluding us from examining this hypothesis. 

The most important finding of this study is that the ICD-9 diagnostic rates were lower by an average of 0.6% in depressive disorders and 0.3% in anxiety states for colorectal cancer patients than noncancer patients. In particular, the newly diagnosed colorectal cancer patients had significantly lower diagnostic rates of depressive and anxiety disorders than the noncancer sample in most years. Because a diagnosis of colorectal cancer is associated with a life-threatening condition, this result suggests the possibility of under-recognition of depressive and anxiety disorders in these patients. The finding can be explained by multiple reasons. It is not uncommon to regard the feeling of depression as a “normal reaction” to a cancer diagnosis. The elderly may be particularly reticent about depression because they have limited knowledge about it [13] and are vulnerable to the “fallacy of good reasons” [14]: attributing depressive symptoms to “normal aging,” cancer or other comorbidity and thus considering treatment not helpful. The same notion can also influence oncologists who may consider depressive symptoms secondary to cancer, thus needing less medical attention, or have neither the time nor financial incentive to treat depression. We used the physician and supplier data file to further examine the diagnostic rates by provider specialty. We found that for colorectal cancer patients, more than 90% of depressive and anxiety diagnoses were provided by primary care and other physicians, while less than 4% of depressive diagnosis and less than 8.2% of anxiety diagnoses were provided by colorectal surgeons and oncologists. There is no evidence that oncologists prescribe psychiatric medication more frequently to patients without a psychiatric diagnosis than do other physicians. Clearly, surgeons and onologists diagnosed cancer patients' depression at a lower rate. Since cancer patients often visit colorectal surgeons and oncologists following a cancer diagnosis instead of family physicians, their chance of having depression diagnosed may be reduced. 

The study outcome indicated some ethnic differences and thus raises concerns about the adequate identification of depression in minority groups. There is an assumption that African-American cancer patients are more depressed due to relatively low socioeconomic status and more serious cancer conditions. Existing literature has established a number of reasons for the underidentification of depression among healthy or outpatient minority population, for example, culturally specific depressive symptoms that are not readily recognizable, the underreporting of mental distress by nonwhite persons, or lack of access to the medical care system [15, 16]. Ethnic difference observed in this study slightly favors a higher diagnostic rate in white patients. Perhaps, because minority cancer patients tend to dwell on physical symptoms that cannot be easily differentiated from cancer symptoms [17], clinical identification of depression is especially challenging for this group of patients. Other results confirmed the existing study findings. Female colorectal cancer patients appeared to be more vulnerable than men to colorectal cancer-related stress as they are to depression in general. Patients aged 85 or older likely had severer comorbid conditions and hence may have been more vulnerable to depression than the young-old patients between the ages of 65 and 74. These findings emphasize the importance of identifying and treating depression in females and the old-old in the elderly colorectal cancer patients. 

Overall, the findings shed light, for the first time, on the ICD-9 psychiatric diagnoses of colorectal cancer patients. They suggest that depressive symptoms in colorectal cancer patients deviate from typical symptoms of major depression and adjustment disorder with depressed mood and are difficult to discern based on current diagnostic criteria. In this study, more than 70% of patients diagnosed for depressive disorders received a diagnosis of depressive disorder NOS in both colorectal cancer patients and noncancer patients. The finding is at odds with the conventional wisdom that fewer cases cannot be diagnosed specifically. It alerts us to the possibility that depressive symptoms in medical patients may be atypical and do not exactly fit the criteria for specific depressive disorders, therefore, they are likely to be labeled as depressive disorder NOS if they are clinically recognized. Because Medicare data are collected for billing purposes rather than research, there is potential for systematic and random bias in coding a diagnosis of depressive disorders automatically as depressive disorder NOS [12]. This reason alone, however, is not sufficient. The issue is particularly relevant to the elderly who tend to underreport depression [13] and nonwhite minority patients who may manifest depression symptoms differently [16, 17]. Lack of attention to depression among colorectal surgeons and oncologists is also worthy of note. The findings strongly recommend a concerted effort to be made to investigate depressive and anxiety symptoms in these cancer patients. This effort will help to educate oncologists and other medical care professionals on how to readily detect psychiatric symptoms and provide prompt referral or treatment to enhance the length and quality of life of these cancer patients and reduce cancer disparities. 

Although the study findings were strengthened by the inclusion of a large portion of colorectal cancer cases in the U.S. and a large noncancer cohort, they have limitations. First, the diagnostic rates were taken from the claim data that approximate—but are not identical to—the clinical diagnosis. Some patients may have been treated with psychiatric medication without a formal diagnosis or sought medical care outside the Medicare system. Second, the study is observational. The diagnosed depressive and anxiety disorders in this cancer population can be due to causes other than colorectal cancer, especially because of the prevalence of comorbidity in this elderly population. Third, the relatively small size of the previously diagnosed sample might have affected the power to detect any significant difference between this sample and the noncancer sample. Finally, a large number of comparisons conducted in this study increases the chance of Type I error. Isolated findings such as a lower depressive diagnostic rate in 1999 in patients with other ethnicity may be at a risk of Type I error. Caution should be exercised in the interpretation of the findings.

## Figures and Tables

**Figure 1:**
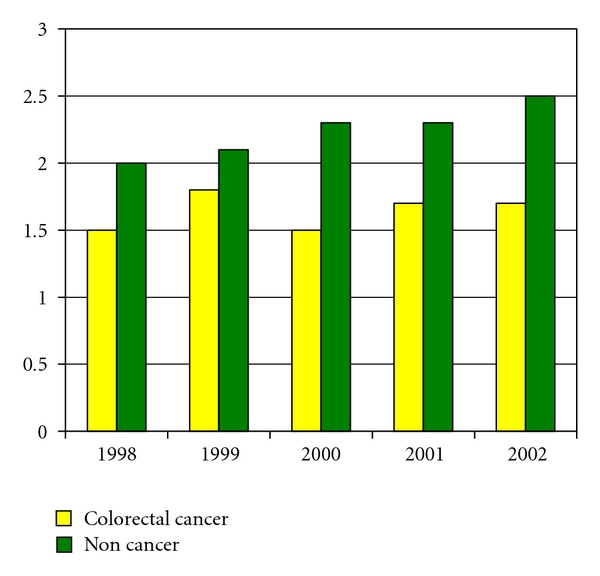
Comparison between a Noncancer Sample and a Colorectal Cancer Sample: Depressive Disorders.

**Table 1:** Descriptive statistics of the colorectal cancer and noncancer samples.

Demographic Variable	Cancer Sample (*N* = 56,182)		Noncancer Sample (*N* = 265,382)	
	*N*	%	*N*	%
Age				
Young-old (65–74)	23,974	42.7	112,702	42.5
Mid-old (75–84)	23,051	41	105,027	39.6
Old-old (≥85)	9,157	16.3	47,653	17.9

Gender				
Male	26,165	46.6	98,061	37
Female	30,017	53.4	167,321	63

Race				
White	47,567	84.7	219,821	82.8
African American	4,311	7.7	19,554	7.4
Hispanic	747	1.3	6,252	2.4
Asian	1,630	2.9	7,877	3
North American Native	68	0.1	767	0.3
Others	1,652	2.9	9,869	3.7
Unknown	207	0.4	1,242	0.5

Cancer Stage				
Stage 0	4,718	8.4		
Stage I	15,048	26.8		
Stage II	16,173	28.8		
Stage III	12,042	21.4		
Stage IV	5,490	9.8		
Unknown	2,711	4.8		

Year of Cancer Diagnosis or Medicare Enrollment of Noncancer Sample				
1998	7308	13	142,696	18.2
1999	7046	12.5	150,447	19.2
2000	13811	24.6	155,383	19.8
2001	13819	24.6	162,300	20.7
2002	14198	25.3	174,372	22.2

**Table 2:** Diagnostic rates of anxiety and depressive disorders and comparisons between noncancer and colorectal cancer patients with or without a previous cancer diagnosis (1998–2002).

Diagnostic Category	Colorectal Cancer			Noncancer
	Newly	Previously	Full	Sample (%)
	Diagnosed (%)	Diagnosed (%)	Sample (%)	(being compared)
1998	*N* = 6045	*N* = 1263	*N* = 7308	*N* = 142696
Depressive disorders	93 (1.5)*	18 (1.4)	111 (1.5)	2789 (2.0)
Major depression	25	4	29	647
Neurotic depression	10	1	11	301
Depression NOS	65	14	79	1973
Adjustment disorder w depressive mood	3	0	3	112
Anxiety states	54 (0.9)	7 (0.6)	61 (0.8)	1624 (1.1)

1999	*N* = 5760	*N* = 1286	*N* = 7046	*N* = 150447
Depressive disorders	107 (1.9)	19 (1.5)	126 (1.8)	3119 (2.1)
Major depression	23	1	24	618
Neurotic depression	8	1	9	298
Depression NOS	75	16	91	2324
Adjustment disorder w depressive mood	6	1	7	100
Anxiety states	72 (1.3)	15 (1.2)	87 (1.2)	1852 (1.2)

2000	*N* = 12519	*N* = 1292	*N* = 13811	*N* = 155383
Depressive disorders	177 (1.4)***	24 (1.9)	201 (1.5)	3500 (2.3)
Major depression	34	5	39	642
Neurotic depression	13	1	14	349
Depression NOS	135	20	155	2637
Adjustment disorder w depressive mood	6	1	7	32
Anxiety states	113 (0.9)***	15 (1.2)	128 (0.9)	2122 (1.4)

2001	*N* = 12400	*N* = 1419	*N* = 13819	*N* = 162300
Depressive disorders	209 (1.7)***	30 (2.1)	239 (1.7)	3782 (2.3)
Major depression	31	2	33	626
Neurotic depression	16	1	17	336
Depression NOS	168	28	196	2948
Adjustment disorder w depressive mood	8	1	9	134
Anxiety states	131 (1.1)**	15 (1.1)	146 (1.1)	2192 (1.4)

2002	*N* = 12554	*N* = 1644	*N* = 14198	*N* = 174372
Depressive disorders	204 (1.6)***	41 (2.5)	245 (1.7)	4340 (2.5)
Major depression	28	3	31	674
Neurotic depression	17	5	22	411
Depression NOS	166	35	201	3439
Adjustment disorder w depressive mood	7	1	8	140
Anxiety states	138 (1.1)***	20 (1.2)	158 (1.1)	2682 (1.5)

*P ≤ .05. **P ≤ .01. ***P ≤ .0001.

**Table 3:** Diagnostic rates of anxiety and depressive disorders by age, gender, and ethnicity.

	1998	1999	2000	2001	2002
Depressive Disorders					
Age:					
Young-old (65–74; being compared)	0.99	1.40	1.22	1.52	1.50
Mid-old (75–84)	1.63*	1.78	1.43	1.66	1.81
Old-old (≥85)	2.32**	2.55*	1.97**	2.32*	1.97
Sex:					
Male	0.94	1.10	0.94	1.12	1.27
Female	2.01***	2.39***	1.89***	2.27***	2.13***
Race:					
White (being compared)	1.67	1.88	1.53	1.82	1.80
Black	0.38*	2.10	0.98	1.05*	1.09
Others	0.90	0.40***	0.88	1.21	1.49

Anxiety Disorders					
Age:					
Middle (being compared)	0.83	1.08	0.92	1.12	1.21
Young	0.81	1.33	0.88	1.16	1.27
Old	0.97	1.57	1.16	1.06	0.86
Sex:					
Male	0.56	0.82	0.61	0.83	0.71
Female	1.10**	1.65**	1.23***	1.38**	1.57***
Race:					
White (being compared)	0.90	1.26	0.99	1.17	1.21
Black	0.57	0.96	0.79	1.15	1.00
Others	0.54	1.59	0.44	0.27	0.75

*P ≤ .05. **P ≤ .01. ***P ≤ .001.
